# Vasorelaxant Mechanism of Herbal Extracts from *Mentha suaveolens*, *Conyza canadensis*, *Teucrium polium* and *Salvia verbenaca* in the Aorta of Wistar Rats

**DOI:** 10.3390/molecules27248752

**Published:** 2022-12-09

**Authors:** Jamila El-Akhal, Andreia P. Oliveira, Rachid Bencheikh, Patrícia Valentão, Paula B. Andrade, Manuela Morato

**Affiliations:** 1 REQUIMTE/LAQV, Laboratory of Pharmacology, Department of Drug Sciences, Faculty of Pharmacy, University of Porto, R. Jorge Viterbo Ferreira, 4050-313 Porto, Portugal; 2 REQUIMTE/LAQV, Laboratório de Farmacognosia, Departamento de Química, Faculdade de Farmácia, Universidade do Porto, R. Jorge Viterbo Ferreira, 4050-313 Porto, Portugal; 3 Microbien Biotechnology and Bioactive Molecules Laboratory, Faculty of Sciences and Technologies, Sidi Mohamed Ben Abdellah University, B.P. 2202, Fez 30000, Morocco

**Keywords:** plant extract, aorta, vasorelaxation, muscarinic receptors, NO, COX products, *Mentha suaveolens*, *Conyza canadensis*, *Teucrium polium*, *Salvia verbenaca*

## Abstract

*Mentha suaveolens* (MS)*, Conyza canadensis* (CC)*, Teucrium polium* (TP) and *Salvia verbenaca* (SV) are used in Morocco to treat hypertension. Our aim was to characterize the composition and vasoreactivity of extracts of MS, CC, TP and SV. The chemical compositions of aqueous extracts of MS, SV and TP, and of a hydromethanolic extract of CC, were identified by HPLC-DAD. The vasoreactive effect was tested in rings of the thoracic aorta of female Wistar rats (8–14 weeks-old) pre-contracted with 10 µM noradrenaline, in the absence or presence of L-NAME 100 µM, indomethacin 10 µM or atropine 6 µM, to inhibit nitric oxide synthase, cyclooxygenase or muscarinic receptors, respectively. L-NAME and atropine decreased the vasorelaxant effect caused by low concentrations of MS. Atropine and indomethacin decreased the vasorelaxant effect of low concentrations of SV. High concentrations of MS or SV and the effect of SV and TP were not altered by any antagonist. The activation of muscarinic receptors and NO or the cyclooxygenase pathway underlie the vasorelaxant effect of MS and SV, respectively. Neither of those mechanisms underlines the vasorelaxant effect of CC and TP. These vasorelaxant effect might support the use of herbal teas from these plants as anti-hypertensives in folk medicine.

## 1. Introduction

A plant-based diet has been suggested to have a protective effect on the cardiovascular system [1,2], with several studies showing a potential benefit related to hypertension [3,4]. Polyphenols are one of the plant constituents that have been more studied on this context, and evidence supports their antihypertensive activity [5], namely through promoting endothelial-dependent and -independent vasorelaxation and improving lipid profile, antioxidant defense and mitochondrial function [2,5,6]. In addition to their use in folk medicine, plants are a useful matrix for the identification of phytochemical compounds further optimized by the pharmaceutical industry [7,8]. Considering its geographical and climate diversity, Morocco provides a large botanical treasure, being an important source of potentially therapeutic plants for the treatment of cardiovascular diseases [9]. Some of the medicinal plants that grow wildly in Morocco and are commonly used in folk medicine for cardiovascular conditions, such as hypertension, are *Mentha suaveolens* Ehrh. (MS), *Conyza Canadensis* L. Cronquist (CC), *Salvia verbenaca* L. (SV) and *Teucrium polium* L. (TP).

MS is a perennial herb that grows wildly in the wet areas of Morocco. It belongs to the Lamiaceae family, grows up to 100 cm in height and has a sickly-sweet scent. It was reported that MS has hypotensive activity [10]. When administered through an IV bolus, methanolic (20–200 mg/kg) and dichloromethanolic (1.1–10 mg/kg) extracts of MS reduced the blood pressure and heart rate of urethane-anaesthetized normotensive rats, and the dichloromethanolic extract also prevented hypertension induced by noradrenaline [11]. Moreover, rotundifolone, a major constituent of the essential oil of *Mentha villosa*, causes hypotension in rats through a non-specific muscarinic receptor stimulation and vasodilatation [12]. The chemical composition of the essential oil of African MS [13,14] has revealed its richness in piperitenone oxides, menthone, pulegone, germacrene D and borneol [14].

CC belongs to the well-known family of the Asteraceae. It is an annual, biennial or perennial plant that grows in very damp ground, submerged in fresh water. CC rarely shrubs, growing 1–2 m high. It grows spontaneously in several regions of Morocco, where it is popular for the treatment of gastrointestinal symptoms, most commonly diarrhea and dysentery, and as a diuretic agent [15]. The aerial parts of CC are mostly rich in limonene [16]. Recently, it has been reported that the acetone and ethyl acetate extracts of CC inhibited the production of nitric oxide (NO) by mouse leukemic macrophage cell line RAW 264.7 but at cytotoxic levels (10–100 mg/mL) [17].

The composition and biological activities of SV have been reviewed recently [18]. SV is a perennial herbaceous plant indigenous to the Mediterranean countries. It grows to 0.6 m, and it is in flower from June to September, while the seeds ripen from July to October. It prefers dry or moist soil and can tolerate drought. SV is used for several health problems including hypertension and as a decoction/infusion [19]. SV is rich in phenolic compounds [20], namely methyl carnosate and rosmarinic acid. Its essential oil is rich in fatty acids and carbonylic compounds [21].

TP is a perennial shrub, 20–50 cm high, that can be found widely distributed in the dry and stony places of the hills and deserts of almost all Mediterranean countries, South-West Asia, Europe and North Africa. The aerial parts and the roots of TP have been used for the treatment of hypertension, pain, inflammation, diabetes, rheumatisms [22,23]. An ethanolic extract of TP (100–400 mg/kg, IV) decreased high blood pressure, but not heart rate, induced by angiotensin II infusion in rats [24]. The major compounds found in the methanolic extract of TP are synaptic acid and eugenol [25], while its essential oil is rich in α-pinene, cis-verbenol and myrtenal [25,26].

In this study, we aimed to characterize the composition of extracts of MS, CC, SV and TP and to test their functional effect as vasoactive extracts using the rat aorta.

## 2. Results

**Figure 1:**
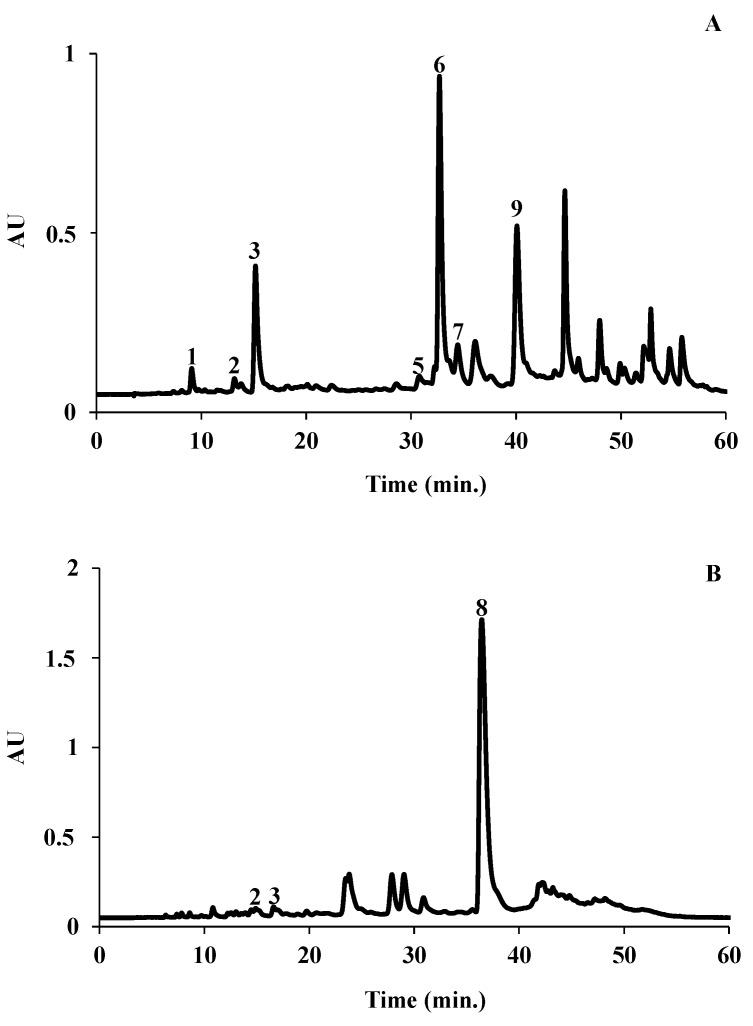
HPLC-DAD phenolic profile of the hydromethanolic extract of *C. Canadensis* (**A**) and of the aqueous extract of *M. suaveolens* (**B**). Detection at 320 nm. (1) 3-*O*-Caffeoylquinic acid, (2) 4-*O*-caffeoylquinic acid, (3) 5-*O*-caffeoylquinic acid, (5) 3,4-di-*O*-caffeoylquinic acid, (6) 3,5-di-*O*-caffeoylquinic acid, (7) 3,4-di-*O*-caffeoylquinic acid, (8) rosmarinic acid and (9) 4,5-di-*O*-caffeoylquinic acid.

The presence of phenolic acids, flavonoids, tannins and alkaloids was screened using general reactions. Phenolic compounds were found in all samples (Table 1). On the other hand, the presence of tannins was only confirmed in the extract from CC, and alkaloids were not detected in any extract (Table 1). The phenolic composition of the extracts was analyzed using HPLC–DAD, and several phenolic acids were identified (Figure 1 and Table 2).

Qualitative and quantitative differences were noticed amongst the four plant extracts (Table 2). The aqueous extracts of MS and CC contained the highest amount of the identified phenolic compounds (25.90 and 11.96 mg/g of dry extract, respectively). Rosmarinic acid was the major compound identified in the aqueous extract of MS (19.80 mg/g of dry sample). The aqueous extract from SV (Lamiaceae) also contained rosmarinic acid but in a much lower amount (0.11 mg/g of dry sample). In this extract, the presence of 3-*O*- and 4-*O*-caffeoylquinic acids was also detected, the last being the most abundant compound (Table 2).

Concerning the aqueous extract of TP, only the presence of 3-*O*-caffeoylquinic and isoferulic acids was detected, the first being the predominant (Table 2).

The hydromethanolic extract of CC displayed higher diversity, with seven hydroxycinnamic acids being identified (Table 2). This extract’s predominant phenolic acid was 3,5-di-*O*-Caffeoylquinic acid (4.17 mg/g of dry sample), followed by 4,5-di-*O*-caffeoylquinic and 5-*O*-caffeoylquinic acids (3.31 and 2.68 mg/g of dry sample, respectively) (Table 2).

**Figure 2:**
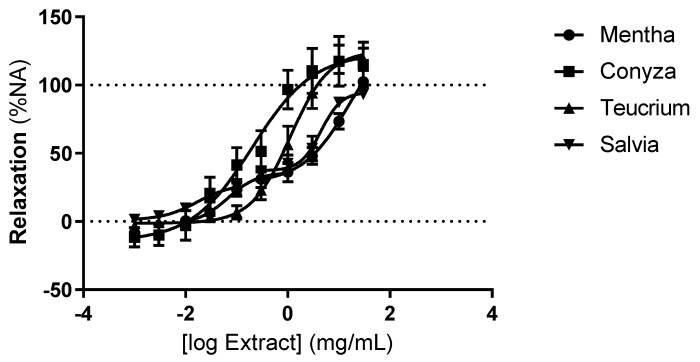
Concentration-response curves of the relaxant effect of the extracts of *M. suavoelens* (MS; circle), *C. canadensis* (CC; square), *T. polium* (TP; triangle) and *S. verbenaca* (SV; inverted triangle) on the rat thoracic aorta. Results expressed as % of the contraction induced by noradrenaline 5 μM. n = 5–7 rats in each group.

The four extracts caused a marked relaxation of the rat thoracic aorta precontracted with noradrenaline (Figure 2) with no difference in the maximum effect between the four extracts (Figure 2 and Table 3). The profiles of the relaxant effect were different among the extracts tested. CC and TP caused simple concentration-dependent relaxation of the rat thoracic aorta (Figure 2), while the relaxant effect of MS and SV showed a two-step profile (Figure 2). This was reflected in a higher EC_50_ for MS and SV when compared with the EC_50_ of CC and TP (MS 5.22 ± 1.96 mg/mL; SV 4.96 ± 0.82 mg/mL; CC 0.30 ± 0.13 mg/mL; TP 1.30 ± 0.35 mg/mL; *p* < 0.05).

**Figure 3:**
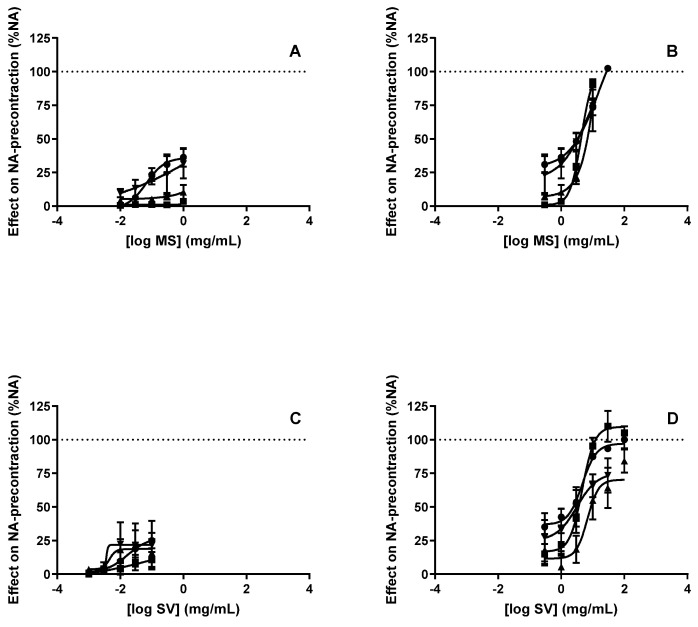
Concentration-response curves for the lower (**A**) and higher (**B**) concentrations of the *M. suaveolens* (MS) extracts and for the lower (**C**) and higher (**D**) concentrations of the *S. verbenaca* (SV), in the absence (control condition, circles) and in the presence of L-NAME 100 µM (squares), Atropine 6 µM (triangle) or Indomethacin 10 µM (inverted triangle), on the rat thoracic aorta. Results expressed as % of the contraction induced by noradrenaline 5 μM. n = 5–7 rats in each group.

Incubation of the rat thoracic aorta rings with L-NAME 100 µM or with atropine 6 µM almost abolished the vasorelaxant effect caused by the lower concentrations of the MS extract tested but had no effect on the vasorelaxation induced by the higher concentrations (Figure 3A,B and Table 3). Indomethacin 10 μM did not alter the vasorelaxation induced by MS (any concentration) in the rat thoracic aorta (Figure 3A,B and Table 3).

The vasorelaxant effect induced by lower concentrations of the SV extract tested was decreased by atropine 6µM and indomethacin 10 μM but not by L-NAME 100 µM, although none of these drugs altered the effect of the higher concentrations tested (Figure 3C,D and Table 3).

**Figure 4:**
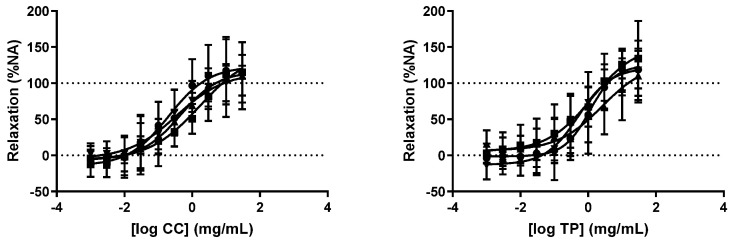
Concentration-response curves for C. canadensis (CC; left) and T. polium (TP; right) in the absence (control condition, circles) and in the presence of L-NAME 100 µM (squares), Atropine 6 µM (triangle) or Indomethacin 10 µM (inverted triangle), on the rat thoracic aorta. Results expressed as % of the contraction induced by noradrenaline 5 μM. n = 5–7 rats in each group.

The concentration-response curves for the vasorelaxant effect of CC or TP were not altered by any of the antagonists tested (Figure 4 and Table 3).

## 3. Discussion

Our study shows that the aqueous extracts of MS, SV and TP and the hydromethanolic extract of CC caused a marked relaxation of the rat thoracic aorta. The profile of the vasorelaxant effect was different among the four plants; while CC and TP caused a typical concentration-response relaxation of the rat thoracic aorta, MS and SV showed a two-step concentration-response vasorelaxation. The mechanisms underlying that effect were characteristic of each plant.

The use of plant extracts, as well as other alternative forms of medical treatments, for the prevention and treatment of diseases has regained popularity in recent years, because they contain various bioactive constituents with health-stimulating properties. In particular, they contain polyphenolic compounds, such as flavonoids and tannins. Therefore, in order to establish a possible relationship between the compounds present in the extracts and their biological potential, the phenolic composition of the extracts was analyzed by HPLC–DAD, and several phenolic acids were identified. The results of the phytochemical analysis of these medicinal plants show the presence of polyphenols in the four extracts. Moreover, only the CC extract was found to contain tannins, and none of the extracts contained alkaloids. Many studies report the beneficial impact of polyphenol consumption in reducing the risk of some chronic diseases such as cardiovascular diseases [27].

Rosmarinic acid was the major compound identified in the aqueous extract of MS, which is in accordance with other Lamiaceae species [28,29]. As this phenolic acid is considered a taxonomic marker of this botanical family and *Mentha* species are promising sources of this metabolite [28], this result was not a surprise. Recently, Sytar and colleagues [30] characterized the phenolic profile of the methanolic extract from MS leaves and, in addition to other hydroxycinnamic acids that were not found in our extract, described the presence of 5-*O*-caffeoylquinic acid, as verified in our sample. The aqueous extract from SV (other Lamiaceae) also contained rosmarinic acid, but in much lower amounts, and we could also detect the presence of 3-*O*- and 4-*O*-caffeoylquinic acids, the last being the most abundant compound.

In the aqueous extract of TP, we could only detect the presence of 3-*O*-caffeoylquinic and isoferulic acids, the first being the more predominant. Although this is a species of the Lamiaceae family, rosmarinic acid was not detected in our aqueous extract, in accordance with what is described for the ethanolic extract of this species [31]. We do not have a clear explanation for this, but it might be related to the particular geographic conditions of the place where the plant was harvested and the fact that we used an aqueous extract instead of a methanolic extract as usually studied. Those authors also identified the presence of 5-*O*-caffeoylquinic, ferulic and caffeic acids [31], which were not detected in our extract.

The hydromethanolic extract of CC displayed higher diversity, with seven hydroxycinnamic acids being identified. The predominant phenolic acid in this extract was 3,5-Di-*O*-Caffeoylquinic acid, followed by the 4,5-di-*O*-caffeoylquinic and 5-*O*-caffeoylquinic acids, which was in agreement with the results obtained by Fraisse et al. [32] for the hydroethanolic extract. Those authors characterized the caffeoyl derivatives of some species from Asteraceae family, including CC, and, in addition to the most abundant compounds, they found low levels of another dicaffeoylquinic acid (1,5-di-*O*-caffeoylquinic acid), which is also in accordance with our results. To the best of our knowledge, 3-*O*-, 4-*O*- and 3,4-di-*O*-caffeoylquinic acids were identified for the first time in this species. Since the phenolic composition is influenced by the environmental conditions (i.e., temperature, humidity and UV irradiation), by the site of collection and by the growing stage [33], the differences obtained in the phenolic profile are not a surprise.

Interestingly, it was recently reported that rosmarinic acid reduces blood pressure in the angiotensin II-induced model of hypertension [34], an effect that seems to be selective for hypertensive (but not normotensive) animals [35]. As this compound was the major one found in the extract of MS, one could think that it was mainly responsible for the vasorelaxant effect of the aqueous extract of MS. However, the aqueous extract from SV induced a similar vasorelaxation in the rat thoracic aorta, and, although it also contained rosmarinic acid, this was present in a much lower amount. So, it is possible that the vasorelaxant effect of rosmarinic acid is strong enough to be evident even in small quantities. As rosmarinic acid was the only compound identified in the extracts of MS and SV, i.e., those which presented a two-step relaxant effect, it is possible that this compound contributes to their potent vasorelaxant effect. Interestingly, in a recent study using the same experimental model that we used in the present study, it was reported that rosmarinic acid lightened the H_2_O_2_-induced endothelial dysfunction [36]. This is in line with the NO-mediated vasorelaxation that we could observe to underlie the lower part of the concentration-response curve obtained for the aqueous extracts of both MS and SV. Moreover, according to this, it is known that polyphenol intake improves endothelial function and has anti-hypertensive effect via the NO-cGMP pathway [37].

The four extracts tested in this study were rich in caffeoylquinic acids, which have antihypertensive effects through several molecular mechanisms, including antioxidant activity and endothelial-dependent NO production [38]. However, to our knowledge, the particular effect of the different caffeoylquinic acids on vasoreactivity or regulation of blood pressure has not been established. Nonetheless, the obtained results suggest that, at least in part, this class of compounds has a strong vasorelaxant effect.

It is well documented that in the vasculature, the endothelial stimulation of muscarinic M_1_, M_3_ and M_5_ receptors produces a vasorelaxant effect [39]. Nevertheless, considering the fact that the vasorelaxation produced by TP and CC on rat aortic segments was unaffected by atropine, an antagonist of muscarinic receptors [40], one can exclude the possible involvement of the stimulation of muscarinic acetylcholine receptors in the vasodilator responses produced by these two extracts under the current experimental conditions. Interestingly, the vasorelaxation induced by the lower concentrations (0.001–0.3 mg/mL) of MS and SV was markedly blocked by atropine, suggesting the involvement of muscarinic receptors. Similarly to what was observed for the vasorelaxant effect of TP and CC, muscarinic receptors do not seem to contribute to the vasorelaxant effect caused by the highest concentrations (1–30 mg/mL) of MS and SV.

One of the pathways through which the activation of muscarinic receptors causes vasorelaxation is the release of NO. Indeed, it is well established that the vascular endothelium can synthesize and release different relaxant factors such as NO and other endothelium-derived relaxing factors (EDRFs) as PGI2 and EDHF. The activation of endothelial muscarinic receptors releases NO, the major EDRF, which causes vasorelaxation through the activation of guanylate cyclase and the production of cGMP [41,42]. In order to elucidate whether the vasorelaxant effect of the tested plant extracts was mediated through the NO pathway, the isolated rat aortic rings were incubated in the presence of L-NAME, an inhibitor of NOS. Under this condition, the vasodilatory effect caused by lower concentrations of MS was almost abolished, evidencing the role of NO in the associated vasorelaxation. On the contrary, the vasorelaxation caused by the higher concentrations of MS and SV and by TP and were not modified by L-NAME, suggesting that they are NO-independent. Conversely, the cyclooxygenase inhibitor indomethacin only inhibited the vasorelaxation induced by lower concentrations of SV, revealing a particular role for the cyclooxygenase pathway in the vasorelaxation of this extract.

Our study has some limitations. First, we only used female rats (for the purpose of compliance with the 3Rs), and the existence of sex differences in vascular reactivity have been reported, with both androgens [43] and estrogens [44] playing a role. Second, our mechanistic reasoning is based on the ex vivo functional approach that was defined to accomplish the aim of the study, and no molecular analysis was performed in order to deepen the characterization of the mechanisms involved in the vasorelaxant effect of the extracts. Third, although the biological potential of the analyzed extracts could be somewhat associated with the identified compounds, we are aware that extracts are complex mixtures and, as such, other compounds and/or metabolites, as well as putative interactions between the different compounds, could also contribute to the observed relaxant activity. Therefore, further scientific explorations are needed to reveal the exact active compound responsible for the observed biological activity of these four Moroccan plant extracts. We decided to work with the aqueous extracts because the fresh leaves and flowers of these plants are usually consumed in Morocco as herbal teas. However, a preliminary study (data not shown) suggested that the gastrointestinal effect concerning CC was more marked when a hydromethanolic extract was tested.

## 4. Materials and Methods

### 4.1. Chemicals and Preparation of Solutions

All the compounds used for the functional assay were obtained from Sigma-Aldrich (USA), except for NaCl (VWR chemicals, Leuven, Belgium), KCl, CaCl_2_, glucose, NaHCO_3_, ascorbic acid (PanreacQuimica, Castellar del Vallès, Spain), NaH_2_PO_4_×H_2_O, Na_2_EDTA, formic acid and methanol Licrosolv^®^ (MERCK, Darmstadt, Germany), 3-*O*-Caffeoylquinic, 4-*O*-caffeoylquinic, 1,5-di-*O*-caffeoylquinic, 3,4-di-*O*-caffeoylquinic, 3,5-di-*O*-caffeoylquinic, 4,5-di-*O*-caffeoylquinic and isoferulic acids (Chengdu Biopurity Phytochemicals Ltd., Chengdu, China), and 5-*O*-caffeoylquinic and rosmarinic acids (Extrasynthèse, Genay, France).

A Krebs-Henseleit solution was prepared on every day of experiment (mM): NaCl 118, KCl 4.8, CaCl_2_ 2.5, MgSO_4_ 1.2, NaH_2_PO_4_ 1.2, Glucose 11, NaHCO_3_ 25, Na_2_EDTA 0.03, and citric acid 0.3. Acetylcholine and KCl stock solutions were prepared every week and stored at 4 °C. All compounds were dissolved in distilled water except indomethacin, which was dissolved in ethanol. Fresh solutions were prepared for each experiment.

### 4.2. Plant Materials

We used the leaves and flowers of MS, CC, SV and TP, which were collected between March and July 2018 from their natural habitat at Taounate, a northern province of Morocco. The authentication of the four plants was performed based on their macroscopic and microscopic characteristics. The macroscopic identification was performed by Khalid Derraz, a botanist at the Faculty of Sciences and Techniques of Fez, Morocco, while the microscopic authentication was completed by Professor Cioanca Oana from the Grigore T. Popa University of Medicine and Pharmacy, Iasi, Romania. The plants were registered in the herbarium of the Department of Biology, Faculty of Sciences and Technologies of the Sidi Mohamed Ben Abdellah University, Fez, Morocco, under the numbers MA-FSTF 21 for MS, MA-FSTF 33 for CC, MA-FSTF 55 for TP and MA-FSTS 44 for SV.

### 4.3. Preparations of the Extracts

The aerial parts of each plant studied (leaves and flowers together) were cleaned, dried in the shade, and reduced to powder with an electric grinder (Moulinex^®^ 320). Then, aqueous extracts of the leaves and flowers of MS, SV and TP were prepared, as well as a hydromethanolic extract from the leaves and flowers of CC. The aqueous extracts of MS, SV and TP were prepared by macerating 50 g of each powdered material in 500 mL of distilled water for 24 h. The hydromethanolic extract of CC was prepared by macerating 50 g of the dried material with 500 mL of a mixture of water:methanol (70:30), over 24 h. The resulting extracts were filtered, centrifuged, frozen at −20 °C and lyophilized (methanol was evaporated to dryness). The percentage yields based on the dried starting materials were 40%, 35% and 45% for the dried aqueous extracts of MS, TP and SV, respectively, and 28% for the dried hydromethanolic extract of CC. All extracts were stored at 4 °C until chemical and pharmacological analyses took place. Voucher specimens were deposited at the Laboratory of Pharmacology, Faculty of Pharmacy, Porto University (MS2017, CC2017, SV2017 and TP2017).

### 4.4. Phytochemical Screening

A phytochemical screening was performed to check for the presence of phenolic acids, flavonoids, tannins and alkaloids, applying standard tests. To screen for the presence of phenolic compounds, a few drops of sodium hydroxide at 20% were added to 1 mL of each extract, with the aim of observing the intensification of the color [45]. The presence of tannins was confirmed using the gelatin reaction [45]. Briefly, one drop of hydrochloric acid was added to 1 mL of each extract followed by the addition of gelatin solution, drop by drop, to avoid the redissolution of the formed precipitate [45].

Alkaloids were checked using the general alkaloid precipitation tests with Dragendorff’s, Mayer’s and Bertrand’s reagents according to a procedure previously described [46]. A total of 10 mL of hydrochloric acid at 10% was added to each extract, and the resulting solutions were alkalinized with a diluted ammonium solution (1:1) and mixed with 20 mL of ethyl ether. Then, a solution of 10 mL of hydrochloric acid at 10% was mixed with the ether phase, and the acidic solution was divided for test tubes to add the Dragendorff’s, Mayer’s and Bertrand’s reagents [45]. This information was improved in the present version.

### 4.5. Phenolic Compounds Analysis

A total of 20 μL of each extract was analyzed on an HPLC unit (Gilson), using a Spherisorb ODS2 (25.0 × 0.46 cm; 5 μm, particle size) column, according to a described procedure [46]. The solvent system used was a gradient of water-formic acid (19:1) (A) and methanol (B), starting with 5% methanol and utilizing a gradient to obtain 15% B at 3 min, 25% B at 13 min, 30% B at 25 min, 35% B at 35 min, 45% B at 39 min, 45% B at 42 min, 50% B at 44 min, 55% B at 47 min, 70% B at 50 min, 75% B at 56 min and 80% B at 60 min, at a solvent flow rate of 0.9 mL/min. Detection was achieved with a Gilson Diode Array Detector (DAD). Data were processed on Unipoint System software (Gilson Medical Electronics, Villiers le Bel, France). The compounds in each extract were identified by comparing their retention times and UV-vis spectra in the 200–400 nm range with authentic standards, and their quantification was achieved by the absorbance recorded in the chromatograms relative to external standards. Each extract was analyzed in triplicate.

### 4.6. Functional Assay

Female Wistar rats (8–14 weeks-old) were used. Animals were maintained at ICBAS-Porto animal facility, Portugal (approved by the national competent authority: 024159/2017-DGAV). All experiments were performed according to the EU Directive 2010/63/EU for animal experiments and reported in accordance with the ARRIVE guidelines. According to the 3R policy of Russel and Burch, we used only female rats, since those were littermates of male rats already ascribed to another ongoing study; with this approach, no extra breeding was performed for the purpose of the present study.

The animals were housed in conventional cages, under standard conditions of temperature (21 °C), humidity (40–60%) and alternative cycles of light and dark (12 h/12 h). They had ad libitum access to autoclaved tap water and a standard laboratory rodent diet (4RF21, Mucedola S.r.l., Milan, Italy). Enrichment material, such as nesting material and paper tunnels, was provided to all cages. All experiments were performed in accordance with the European Union guidelines for the protection of animals used for scientific purposes (Directive 2010/63/EU and Decision 1999/575/EC) and Portuguese guidelines (Direção-Geral da Alimentação e Veterinária, DGAV, Portugal).

On the day of the functional experiment, the animals were randomly euthanized by decapitation using a small animal guillotine. The thoracic aorta was removed, cleaned of adhering connective tissue and cut into 5 mm length rings. Then, the rings were mounted in organ baths filled with warmed (37 °C) and aerated (95% O_2_, 5% CO_2_) Krebs–Henseleit solution, under a resting tension of 1.5 g. Each aortic ring was allowed to equilibrate for 60 min. After this, the endothelium viability was confirmed by a relaxant effect of acetylcholine 10 µM over a 10 µM noradrenaline-induced precontraction. Only those rings that showed a relaxation higher than 50% of the noradrenaline-induced precontraction were used. The tissue was then washed every 10 min for 30 min. Then, the tissue was contracted with 10 µM noradrenaline, and cumulative concentration-response curves were obtained for each extract in the absence or presence of L-NAME 100 µM (a nitric oxide synthase inhibitor), indomethacin 10 µM (a cyclooxygenase inhibitor) or atropine 6 µM (an antagonist of muscarinic receptors). When used, the antagonists were added to the organ bath 20 min before the second noradrenaline-induced precontraction. Only one experimental condition (control or in the presence of one drug) was randomly tested in each aortic ring. The range of concentrations of the extracts tested showed to be non-cytotoxic in the MTT assay performed in A375 skin cancer cells and in MCF-7 breast cancer cells (data not shown).

### 4.7. Statistical Analysis

The experimental unit was the individual animal. For each functional experiment, the response of each extract (in the absence or presence of any antagonist) was calculated as a percentage of the correspondent stable noradrenaline-induced precontraction. E_max_ (maximum effect) and EC_50_ values (concentration of the extract causing 50% of the maximum response) were calculated using GraphPad Prism software, version 7.00 for Windows. All the values were expressed as mean ± standard error of the mean (SEM). Statistical analysis was preformed using one-way ANOVA followed by Tukey’s multiple comparisons test to compare the E_max_ or the EC_50_ values among the four plants and one-way ANOVA followed by Dunnett’s multiple comparisons test to compare the E_max_ between the effect in the presence of each antagonist and the effect in the control condition (no pretreatment). A *p* value of less than 0.05 was considered statistically significant. Sample size was not calculated, since all the animals were already ascribed to another ongoing study of the research group (Reduction); only the thoracic aorta was used in this study.

## 5. Conclusions

To conclude, our study has shown for the first time that the aqueous extracts of leaves and flowers of MS, SV and TP and the hydromethanolic extract of leaves and flowers of CC possess a potent vasorelaxant effect which might justify their use in folk medicine for the treatment and prevention of cardiovascular diseases such as hypertension. Our results suggest that the activation of muscarinic receptors and NO production contribute to the vasorelaxation induced by MS and that activation of muscarinic receptors and PGI_2_ production contributes to the vasorelaxation induced by SV, particularly at low concentrations. Conversely, high concentrations of MS and SV and the vasorelaxation caused by CC and TP are independent of muscarinic receptors, NO production and the cyclooxygenase pathway.

## Figures and Tables

**Table 1:** Phytochemical screening of extracts from *M. suaveolens* (MS), *C. canadensis* (CC), *S. verbenaca* (SV) and *T. polium* (TP).

Samples	Phenolic Compounds	Tannins	Alkaloids
MS	+	−	−
SV	+	−	−
TP	+	−	−
CC	+	+	−

+, presence; −, absence.

**Table 2:** Phenolic composition of the extracts from the *M. suaveolens* (MS), *C. canadensis* (CC), *S. verbenaca* (SV) and *T. polium* (TP).

Phenolics		mg/g of Dry Extract			
		MS	CC	TP	SV
1	3-*O*-CQA	-	0.30 ± 0.00 (2.51)	0.11 ± 0.00 (91.67)	0.10 ± 0.01 (25.64)
2	4-*O*-CQA	2.13 ± 0.04 (8.22)	0.20 ± 0.00 (1.67)	-	0.18 ± 0.00 (46.15)
3	5-*O*-CQA	3.97 ± 0.20 (15.33)	2.68 ± 0.02 (22.41)	-	-
4	Isoferulicacid	-	-	0.01 ± 0.00 (8.33)	-
5	3,4-di-*O*-CQA	-	0.54 ± 0.01 (4.52)	-	-
6	3,5-di-*O*-CQA	-	4.17 ± 0.19 (34.87)	-	-
7	1,5-di-*O*-CQA	-	0.76 ± 0.04 (6.35)	-	-
8	Rosmarinic acid	19.80 ± 1.08 (76.45)	-	-	0.11 ± 0.00 (28.21)
9	4,5-di-*O*-CQA	-	3.31 ± 0.28 (27.68)	-	-
	Total	25.90	11.96	0.12	0.39

Values are expressed as mean ± standard deviation (and %) of three determinations; CQA, caffeoylquinic acid; nq, not quantifiable.

**Table 3:** Maximum relaxant effect (Emax; %NA) for the four extracts in the absence (control condition) and in the presence of L-NAME 100 µM, Atropine 6 µM or Indomethacin 10 µM, on the rat thoracic aorta.

E_max_ (% NA)	Control	L-NAME	Atropine	Indomethacin
MS-Lower	40.26 ± 6.75	7.50 ± 3.38 *	5.20 ± 2.22 *	41.50 ± 11.50
MS-Higher	101.40 ± 14.67	114.00 ± 25.77	79.16 ± 2.44	75.20 ± 19.09
SV-Lower	35.44 ± 4.37	14.46 ± 6.78	22.89 ± 9.97 *	2.98 ± 10.46 *
SV-Higher	94.95 ± 2.40	114.50 ± 12.18	73.61 ± 15.97	70.30 ± 14.61
TP	122.1 ± 8.80	143.80 ± 17.72	106.10 ± 5.90	123.00 ± 18.61
CC	126.70 ± 19.11	154.30 ± 25.31	123.20 ± 13.37	119.00 ± 20.61

Values are mean ± S.E.M., n = 5–7 rats in each group. * *p* < 0.05 vs. the corresponding control.

## Data Availability

Data is available upon request.

## References

[1] Lopes T., Zemlin A.E., Erasmus R.T., Madlala S.S., Faber M., Kengne A.P. (2022). Assessment of the association between plant-based dietary exposures and cardiovascular disease risk profile in sub-Saharan Africa: A systematic review. BMC Public Health.

[2] Chavez-Castillo M., Ortega A., Duran P., Pirela D., Marquina M., Cano C., Salazar J., Gonzalez M.C., Bermudez V., Rojas-Quintero J. (2020). Phytotherapy for Cardiovascular Disease: A Bench-to-Bedside Approach. Curr. Pharm. Des..

[3] Kamyab R., Namdar H., Torbati M., Ghojazadeh M., Araj-Khodaei M., Fazljou S.M.B. (2021). Medicinal Plants in the Treatment of Hypertension: A Review. Adv. Pharm. Bull..

[4] Verma T., Sinha M., Bansal N., Yadav S.R., Shah K., Chauhan N.S. (2021). Plants Used as Antihypertensive. Nat. Prod. Bioprospect..

[5] Grosso G., Godos J., Currenti W., Micek A., Falzone L., Libra M., Giampieri F., Forbes-Hernandez T.Y., Quiles J.L., Battino M. (2022). The Effect of Dietary Polyphenols on Vascular Health and Hypertension: Current Evidence and Mechanisms of Action. Nutrients.

[6] Kim B., Lee K., Chinannai K.S., Ham I., Bu Y., Kim H., Choi H.Y. (2015). Endothelium-Independent Vasorelaxant Effect of *Ligusticum jeholense* Root and Rhizoma on Rat Thoracic Aorta. Molecules.

[7] Yao J., Weng Y., Dickey A., Wang K.Y. (2015). Plants as Factories for Human Pharmaceuticals: Applications and Challenges. Int. J. Mol. Sci..

[8] Hao D.C., Xiao P.G. (2020). Pharmaceutical resource discovery from traditional medicinal plants: Pharmacophylogeny and pharmacophylogenomics. Chin. Herb. Med..

[9] Jouad H., Haloui M., Rhiouani H., El Hilaly J., Eddouks M. (2001). Ethnobotanical survey of medicinal plants used for the treatment of diabetes, cardiac and renal diseases in the North centre region of Morocco (Fez-Boulemane). J. Ethnopharmacol..

[10] Bozovic M., Pirolli A., Ragno R. (2015). *Mentha suaveolens* Ehrh. (Lamiaceae) Essential Oil and Its Main Constituent Piperitenone Oxide: Biological Activities and Chemistry. Molecules.

[11] Bello R., Calatayud S., Beltran B., Primo-Yufera E., Esplugues J. (2001). Cardiovascular effects of the methanol and dichloromethanol extracts from *Mentha suaveolens* Ehrh. Phytother. Res..

[12] Guedes D.N., Silva D.F., Barbosa-Filho J.M., Medeiros I.A. (2002). Muscarinic agonist properties involved in the hypotensive and vasorelaxant responses of rotundifolone in rats. Planta Med..

[13] Kasrati A., Alaoui Jamali C., Bekkouche K., Spooner-Hart R., Leach D., Abbad A. (2015). Chemical characterization and insecticidal properties of essential oils from different wild populations of *Mentha suaveolens* subsp. *timija* (Briq.) Harley from Morocco. Chem. Biodivers..

[14] Kasrati A., Jamali C.A., Bekkouche K., Lahcen H., Markouk M., Wohlmuth H., Leach D., Abbad A. (2013). Essential oil composition and antimicrobial activity of wild and cultivated mint timija (*Mentha suaveolens* subsp. *timija* (Briq.) Harley), an endemic and threatened medicinal species in Morocco. Nat. Prod. Res..

[15] Healthcare T. (2008). PDR for Herbal Medicines.

[16] Veres K., Csupor-Loffler B., Lazar A., Hohmann J. (2012). Antifungal activity and composition of essential oils of *Conyza canadensis* herbs and roots. Sci. World J..

[17] Adebayo S.A., Ondua M., Shai L.J., Lebelo S.L. (2019). Inhibition of nitric oxide production and free radical scavenging activities of four South African medicinal plants. J. Inflamm. Res..

[18] Mrabti H.N., El Menyiy N., Charfi S., Saber M., Bakrim S., Alyamani R.A., Rauf A., Ali A.M.H., Abdallah E.M., El Omari N. (2022). Phytochemistry and Biological Properties of *Salvia verbenaca* L.: A Comprehensive Review. Biomed Res. Int..

[19] Orch H., Douira A., Zidane L. (2015). Étude ethnobotanique des plantes médicinales utilisées dans le traitement du diabète, et des maladies cardiaques dans la région d’Izarène (Nord du Maroc). J. Appl. Biosci..

[20] Ben Farhat M., Chaouch-Hamada R., Sotomayor J.A., Landoulsi A., Jordan M.J. (2015). Antioxidant properties and evaluation of phytochemical composition of Salvia verbenaca L. extracts at different developmental stages. Plant Foods Hum. Nutr..

[21] Canzoneri M., Bruno M., Rosselli S., Russo A., Cardile V., Formisano C., Rigano D., Senatore F. (2011). Chemical composition and biological activity of *Salvia verbenaca* essential oil. Nat. Prod. Commun..

[22] Bahramikia S., Yazdanparast R. (2012). Phytochemistry and medicinal properties of *Teucrium polium* L. (Lamiaceae). Phytother. Res..

[23] Suleiman M.S., Abdul-Ghani A.S., Al-Khalil S., Amin R. (1988). Effect of Teucrium polium boiled leaf extract on intestinal motility and blood pressure. J. Ethnopharmacol..

[24] Mahmoudabady M., Shafei M.N., Niazmand S., Khodaee E. (2014). The Effects of Hydroalchoholic Extract of *Teucrium polium* L. on Hypertension Induced by Angiotensin II in Rats. Int. J. Prev. Med..

[25] Purnavab S., Ketabchi S., Rowshan V. (2015). Chemical composition and antibacterial activity of methanolic extract and essential oil of Iranian *Teucrium polium* against some of phytobacteria. Nat. Prod. Res..

[26] Nikpour H., Mousavi M., Asadollahzadeh H. (2018). Qualitative and quantitative analysis of *Teucrium polium* essential oil components by GC-MS coupled with MCR and PARAFAC methods. Phytochem. Anal..

[27] Manach C., Mazur A., Scalbert A. (2005). Polyphenols and prevention of cardiovascular diseases. Curr. Opin. Lipidol..

[28] Ferreres F., Bernardo J., Andrade P.B., Sousa C., Gil-Izquierdoa A., Valentão P. (2015). Pennyroyal and gastrointestinal cells: Multi-target protection of phenolic compounds against t-BHP-induced toxicity. RSC Adv..

[29] Gonçalves S., Moreira E., Grosso C., Andrade P.B., Valentão P., Romano A. (2017). Phenolic profile, antioxidant activity and enzyme inhibitory activities of extracts from aromatic plants used in Mediterranean diet. J. Food Sci. Technol..

[30] Sytar O., Hemmerich I., Zivcak M., Rauh C., Brestic M. (2018). Comparative analysis of bioactive phenolic compounds composition from 26 medicinal plants. Saudi J. Biol. Sci..

[31] Vladimir-Knezevic S., Blazekovic B., Kindl M., Vladic J., Lower-Nedza A.D., Brantner A.H. (2014). Acetylcholinesterase inhibitory, antioxidant and phytochemical properties of selected medicinal plants of the Lamiaceae family. Molecules.

[32] Fraisse D., Felgines C., Texier O., Lamaison J.-L. (2011). Caffeoyl derivatives: Major antioxidant compounds of some wild herbs of the Asteraceae family. Food Nutr. Sci..

[33] Tomás-Barberán F.A., Espín J.C. (2001). Phenolic compounds and related enzymes as determinants of quality in fruits and vegetables. J. Sci. Food Agric..

[34] Alegría-Herrera E., Herrera-Ruiz M., Román-Ramos R., Zamilpa A., Santillán-Urquiza M.A., Aguilar M.I., Avilés-Flores M., Fuentes-Mata M., Jiménez-Ferrer E. (2019). Effect of Ocimumbasilicum, Ocimumselloi, and Rosmarinic Acid on Cerebral Vascular Damage in a Chronic Hypertension Model. Biol. Pharm. Bull..

[35] Ferreira L.G., Evora P.R.B., Capellini V.K., Albuquerque A.A., Carvalho M.T.M., Gomes R., Parolini M.T., Celotto A.C. (2018). Effect of rosmarinic acid on the arterial blood pressure in normotensive and hypertensive rats: Role of ACE. Phytomedicine.

[36] Zhou H., Fu B., Xu B., Mi X., Li G., Ma C., Xie J., Li J., Wang Z. (2017). Rosmarinic Acid Alleviates the Endothelial Dysfunction Induced by Hydrogen Peroxide in Rat Aortic Rings via Activation of AMPK. Oxidative Med. Cell Longev..

[37] Hugel H.M., Jackson N., May B., Zhang A.L., Xue C.C. (2016). Polyphenol protection and treatment of hypertension. Phytomedicine.

[38] Zhao Y., Wang J., Ballevre O., Luo H., Zhang W. (2012). Antihypertensive effects and mechanisms of chlorogenic acids. Hypertens. Res..

[39] Harvey R.D. (2012). Muscarinic receptor agonists and antagonists: Effects on cardiovascular function. Handb. Exp. Pharmacol..

[40] Cuthbert A.W. (1963). Some Effects of Atropine on Smooth Muscle. Br. J. Pharmacol. Chemother..

[41] Calver A., Collier J., Vallance P. (1993). Nitric oxide and cardiovascular control. Exp. Physiol..

[42] Rapoport R.M., Murad F. (1983). Agonist-induced endothelium-dependent relaxation in rat thoracic aorta may be mediated through cGMP. Circ. Res..

[43] Lucas-Herald A.K., Touyz R.M. (2022). Androgens and Androgen Receptors as Determinants of Vascular Sex Differences Across the Lifespan. Can. J. Cardiol..

[44] Riedel K., Deussen A.J., Tolkmitt J., Weber S., Schlinkert P., Zatschler B., Friebel C., Muller B., El-Armouche A., Morawietz H. (2019). Estrogen determines sex differences in adrenergic vessel tone by regulation of endothelial beta-adrenoceptor expression. Am. J. Physiol. Heart Circ. Physiol..

[45] Bruneton J., Doc É.T. (2016). Pharmacognosie, Phytochimie, Plants Médicinals.

[46] Oliveira A.P., Valentao P., Pereira J.A., Silva B.M., Tavares F., Andrade P.B. (2009). *Ficus carica* L.: Metabolic and biological screening. Food Chem. Toxicol..

